# Primary retroperitoneal lymph node dissection for clinical stage II seminoma: Comparative analysis against established paradigms using the National Cancer Data Base

**DOI:** 10.1002/bco2.70229

**Published:** 2026-05-18

**Authors:** Josiah K. Low, John E. Musser

**Affiliations:** ^1^ Naval Medical Center San Diego San Diego California USA

**Keywords:** chemotherapy, database, retroperitoneal lymph node dissection, seminoma, testicular cancer

## Abstract

**Objectives:**

This paper aims to perform a comparative, real‐world analysis of outcomes after primary retroperitoneal lymph node dissection (pRPLND) for clinical stage II (CSII) seminoma.

**Patients and Methods:**

Using the National Cancer Data Base, we identified patients with CSII germ cell tumours (GCT) between 2004 and 2022. Cox regression was used for survival analyses. Primary analysis examined overall survival (OS) for patients with CSII seminoma. A sensitivity analysis after energy‐balancing weighting was performed. Secondary analysis examined salvage‐chemotherapy‐free survival (SFS) after pRPLND for CSII seminoma.

**Results:**

Primary analysis included 4661 patients (median follow‐up 6.8 years, IQR 3.6–10.5 years). OS did not differ by primary treatment (vs. chemotherapy—HR1.34, 95% CI 0.93–1.93, *p* = 0.12; versus radiation—1.02, 0.65–1.59, *p* = 0.95). Sensitivity analysis results were similar. Secondary analysis demonstrated estimated 2‐year salvage chemotherapy‐free survival rates of 70%, 45% and 36% for cN1, cN2 and cN3, respectively. Unaccounted bias should be considered given the retrospective nature of the underlying data.

**Conclusion:**

OS after pRPLND for CSII seminoma appears comparable to alternatives. Risk of receiving salvage chemotherapy increases with clinical disease extent.

## 1 INTRODUCTION

Primary retroperitoneal lymph node dissection (pRPLND) has been an established treatment for clinical stage (CS) IIA/B nonseminomatous germ cell tumour (nsGCT) for decades. By contrast, the treatment paradigm for CS IIA/B seminoma has, until relatively recently, consisted of chemotherapy and/or radiation. While these options afford excellent cure rates, they are associated with long‐term adverse effects, including secondary malignancies and toxicities.^1^

The 2024 update to the European Association of Urology Testicular Cancer Guidelines and 2025 update to the National Comprehensive Cancer Network (NCCN) Testicular Cancer Guidelines added pRPLND as a treatment option for CS IIA/B seminoma.^2^, ^3^ Until 2023, supporting data for this approach were limited to retrospective case series and database studies.^4^, ^5^, ^6^, ^7^, ^8^ Results of the Surgery in Early Metastatic Seminoma (SEMS) trial provided the first prospective data in support of pRPLND for CS IIA/B seminoma, followed by data from the Primary Retroperitoneal Lymph Node Dissection in Patients with Seminomatous Testicular Germ Cell Tumors with Clinical Stage IIA/B (PRIMETEST) and COlogne Trial of Retroperitoneal Lymphadenectomy In Metastatic Seminoma (COTRIMS) trials.^9^, ^10^, ^11^ Absent from existing literature, however, is any comparative analysis of pRPLND against chemotherapy and radiation in the treatment of CS IIA/B seminoma.

The purpose of this study was to conduct the first comparative analysis of pRPLND for CS II seminoma against other established treatment paradigms for CS IIA/B seminoma. Due to the challenges of obtaining prospective, randomized, head‐to‐head data for a low‐incidence disease, we utilized the National Cancer Data Base (NCDB) to facilitate this analysis.

## 2 PATIENTS AND METHODS

### 2.1 Data Source

The NCDB is a national clinical oncology database that captures institutionally based, patient‐level data. Included are over 1500 Commission on Cancer‐accredited institutions and over 30 million patients, with capture of approximately 70% of all newly diagnosed cancers within the USA. We used data from the NCDB Testis Cancer Participant User File (PUF) from 2004 to 2022.

### 2.2 Study Design

Patients with testicular tumours (International Classification of Diseases for Oncology, 3rd edition [ICD‐O‐3] topography codes C62) and ICD‐O‐3 histology codes for seminoma (9016 and 9062) were identified. Patients with CS II disease were identified based on American Joint Committee on Cancer Stage Manual staging info.^12^ Patients missing orchiectomy pathology or primary treatment data were excluded. pRPLND was defined based on documented regional node surgery combined with two sequencing variables that specified receipt of RPLND prior to chemotherapy/radiation or receipt of RPLND alone.

Our primary analysis compared overall survival (OS) between RPLND, chemotherapy and radiation in the primary treatment setting for CS II seminoma. Our secondary analysis assessed salvage chemotherapy‐free survival (SFS) among seminoma patients who underwent pRPLND. Of note, no NCDB variable exists to explicitly distinguish salvage and adjuvant treatments. Based on recurrence data from the SEMS, PRIMETEST and COTRIMS trials, a conservative cutoff of 2 months was selected, with treatment earlier presumed to be adjuvant and treatment later presumed to be salvage. Patients with presumed adjuvant treatment were excluded from this analysis.

### 2.3 Covariates

Patient covariates included age, year of diagnosis, Charlson–Deyo score, race, insurance status, annual income quartile, education level and distance between location of residence and treating hospital. Disease covariates included primary tumour size (<3 cm or ≥3 cm), orchiectomy margin status, pathological T stage (pT), clinical N stage (cN) and, for patients who underwent pRPLND, pathological N stage (pN). Follow‐up duration for OS was determined by the interval between diagnosis date until either death or date of last contact. Follow‐up duration for SFS was determined by the interval between diagnosis and receipt of presumed salvage (or date of last contact for cases without documented salvage).

### 2.4 Statistical analysis

For our primary analysis, baseline patient and disease characteristics were compared by primary treatment modality. Analysis of variance (ANOVA) was used to identify significant differences in age. Chi‐squared or Fisher's exact tests were used for statistical testing for the remaining characteristics.

Initial univariate Cox regression analysis was performed on all covariates to identify potentially significant covariates for OS in CS II seminoma. Significant patient covariates, in addition to all disease characteristics, were subsequently used to perform multivariable Cox regression analysis for OS. Sensitivity analysis to validate our multivariable model was performed. Energy‐balancing weighting, an approach shown to reduce bias in estimating treatment effect compared to propensity‐score weighting methods, was used to calculate covariate weights.^13^ Weighted multivariable Cox regression analysis was then performed and compared against our initial unweighted model. Kaplan–Meier curves were generated for OS by primary treatment modality.

For the secondary analysis, baseline characteristics were compared with ANOVA, Chi‐squared and Fisher's exact tests. Multivariable Cox regression models using disease characteristics and patient characteristics found to be significant on initial univariate Cox regressions were created. Kaplan–Meier curves were generated.

All statistical analysis was performed using R (R Core Team, Vienna, Austria).^14^, ^15^, ^16^, ^17^, ^18^ All tests were two‐sided with *p* < 0.05 considered statistically significant. An institutional review board waiver was granted for this study based on established policies on usage of deidentified data.

### 2.5 Role of the funding source

No dedicated funding was provided for this study.

## 3 RESULTS

### 3.1 Primary analysis

**FIGURE 1:**
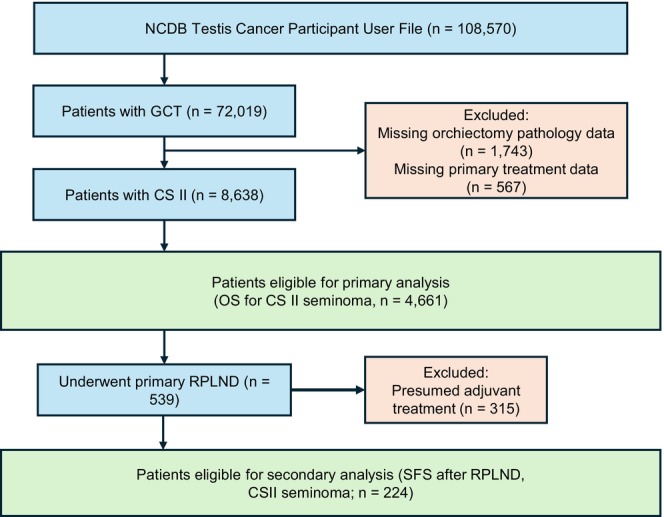
Study cohort selection flow diagram (STrengthening the Reporting of OBservational studies in Epidemiology style).

**FIGURE 2:**
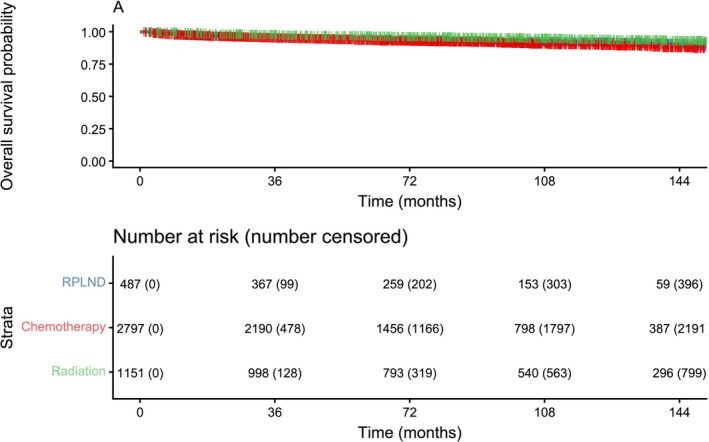
Kaplan–Meier estimated overall survival curves for clinical stage II (CS II) seminoma by primary treatment. Curve truncated after <10% of patients remain at risk.

From 2004 to 2022, 4661 patients were diagnosed with CS II seminoma. pRPLND was performed in 539 of these patients (Figure 1). Increased utilization of pRPLND was observed in more recent years (Figure S1). OS was 94% at median follow‐up of 6.8 years (IQR 3.6–10.5 years). Patients who underwent primary chemotherapy or radiation differed significantly in every patient and disease characteristic except for residential setting (Table 1). Expected 2‐year OS for all primary treatment modalities was high, at 96%, 96%, and 98% for pRPLND, chemotherapy and radiation, respectively (Figure 2). These rates remained high over time, with expected OS rates at median follow‐up of 94%, 92% and 96%, respectively. Preliminary univariable Cox regression results are reported in Table S1. Multivariable Cox regression did not show a significant difference in OS between primary treatment modalities (Table 2). This remained the case in sensitivity analysis with weighted multivariable Cox regression (Tables S2 and S3). Increased age, Charlson–Deyo score of 1 or 2, income below highest quartile and pT4 were associated with reduced OS. Private insurance was associated with increased OS (Table 2).

**TABLE 1:** Overall baseline patient characteristics.

		RPLND	Chemo	RT	*p* value
		*n* = 539	*n* = 2951	*n* = 1171	
Age (median [IQR])		42 [34–50]	38 [31–46]	37 [31–46]	<0.0001
Year of diagnosis	2004–2010	99 (18%)	674 (23%)	465 (40%)	<0.0001
	2011–2016	214 (40%)	1125 (38%)	484 (41%)	
	2017–2022	226 (42%)	1152 (39%)	222 (19%)	
Charlson–Deyo score	CD0	456 (85%)	2695 (91%)	1094 (93%)	0.00050
	CD1	64 (12%)	204 (7%)	58 (5%)	
	CD2	11 (2%)	33 (1%)	12 (1%)	
	CD3	8 (1%)	19 (1%)	7 (1%)	
Race	White	440 (82%)	2286 (77%)	975 (83%)	0.022
	Black	23 (4%)	132 (4%)	42 (4%)	
	Hispanic	59 (11%)	382 (13%)	105 (9%)	
	Other/Unknown	17 (3%)	151 (5%)	49 (4%)	
Insurance	Medicaid/Medicare/Other Government	122 (23%)	614 (21%)	164 (14%)	<0.0001
	Private	371 (69%)	1946 (66%)	873 (75%)	
	Uninsured/unknown	46 (9%)	391 (13%)	134 (11%)	
Residential category	Metro	435 (81%)	2375 (80%)	976 (83%)	0.059
	Urban	64 (12%)	419 (14%)	143 (12%)	
	Rural	13 (2%)	45 (2%)	20 (2%)	
	Unknown	27 (5%)	112 (4%)	32 (3%)	
Income quartile	>$74 062	184 (34%)	961 (33%)	467 (40%)	<0.0001
	$57 857–$74 062	112 (21%)	647 (22%)	250 (21%)	
	$46 277–$57 856	97 (18%)	529 (18%)	206 (18%)	
	<$46 277	54 (10%)	381 (13%)	123 (11%)	
	Unknown	92 (17%)	433 (15%)	125 (11%)	
Education (% without high school education)	<5.0%	110 (20%)	559 (19%)	276 (24%)	<0.0001
	5.0%–9.0%	146 (27%)	720 (24%)	325 (28%)	
	9.1%–15.2%	118 (22%)	676 (23%)	278 (24%)	
	>15.2%	75 (14%)	575 (19%)	170 (15%)	
	Unknown	90 (17%)	421 (14%)	122 (10%)	
Distance between residence and hospital	<10 miles	196 (36%)	1298 (44%)	575 (49%)	<0.0001
	≥10 miles	256 (47%)	1248 (42%)	481 (41%)	
	Unknown	87 (16%)	405 (14%)	115 (10%)	
Tumour size	<3 cm	235 (44%)	582 (20%)	200 (17%)	<0.0001
	≥3 cm	304 (56%)	2369 (80%)	971 (83%)	
Orchiectomy margin	R0	510 (95%)	2779 (94%)	1149 (98%)	<0.0001
	R1	29 (5%)	172 (6%)	22 (2%)	
Pathologic T stage	pT0	45 (8%)	88 (3%)	2 (0%)	0.00050
	pTis	0 (0%)	2 (0%)	0 (0%)	
	pT1	263 (49%)	1295 (44%)	649 (55%)	
	pT2	176 (33%)	1131 (38%)	414 (35%)	
	pT3	33 (6%)	305 (10%)	51 (4%)	
	pT4	2 (0%)	20 (1%)	1 (0%)	
	pTx	20 (4%)	110 (4%)	54 (5%)	
Clinical N stage	cN1	132 (24%)	629 (21%)	818 (70%)	<0.0001
	cN2	157 (29%)	1037 (35%)	342 (29%)	
	cN3	250 (46%)	1285 (44%)	11 (1%)	
Pathologic N stage	pN1	90 (17%)	( )	( )	( )
	pN2	105 (19%)	( )	( )	
	pN3	143 (27%)	( )	( )	
	pN0	68 (13%)	( )	( )	

Abbreviations: CS II, clinical stage II; RPLND, retroperitoneal lymph node dissection; RT, radiation therapy.

**TABLE 2:** Multivariate Cox regression model—Primary analysis.

		HR	LCI	UCI	*p* value
Age (per 1 year)		1.04	1.03	1.05	<0.0001
Charlson–Deyo score (ref: CD0)	CD1	1.57	1.12	2.19	0.0094
	CD2	4.05	2.47	6.66	<0.0001
	CD3	1.52	0.66	3.46	0.32
Insurance (ref: Medicaid/Medicare/Other Government)	Private	0.51	0.39	0.65	<0.0001
	Uninsured	0.82	0.57	1.16	0.25
Income quartile (ref: >$74 062)	$57 857–$74 062	1.49	1.07	2.07	0.020
	$46 277–$57 856	1.95	1.37	2.77	0.00021
	<$46 277	2.08	1.38	3.13	0.00046
	Unknown	3.05	0.95	9.75	0.060
Education (% without high school education) (ref: <5.0%)	5.0%–9.0%	1.01	0.70	1.45	0.95
	9.1%–15.2%	1.01	0.69	1.48	0.95
	>15.2%	0.89	0.58	1.37	0.60
	Unknown	0.36	0.11	1.19	0.094
Orchiectomy margin (ref: R0)	R1	1.34	0.89	2.02	0.16
Pathologic T stage (ref: pT1)	pT2	1.12	0.87	1.44	0.38
	pT3	1.39	0.97	1.99	0.069
	pT4	4.29	1.69	10.87	0.0021
	pTis	0.00	0.00	Inf	0.99
	pT0	0.84	0.47	1.50	0.54
	pTx	0.89	0.53	1.52	0.68
Clinical N stage (ref: cN1)	cN2	0.98	0.73	1.33	0.92
	cN3	1.32	0.98	1.79	0.070
Primary treatment modality (ref: RPLND)	Chemotherapy	1.34	0.93	1.93	0.12
	Radiation	1.02	0.65	1.59	0.95

Abbreviation: RPLND, retroperitoneal lymph node dissection.

### 3.2 Secondary analysis—SFS for pRPLND in CS II seminoma

**FIGURE 3:**
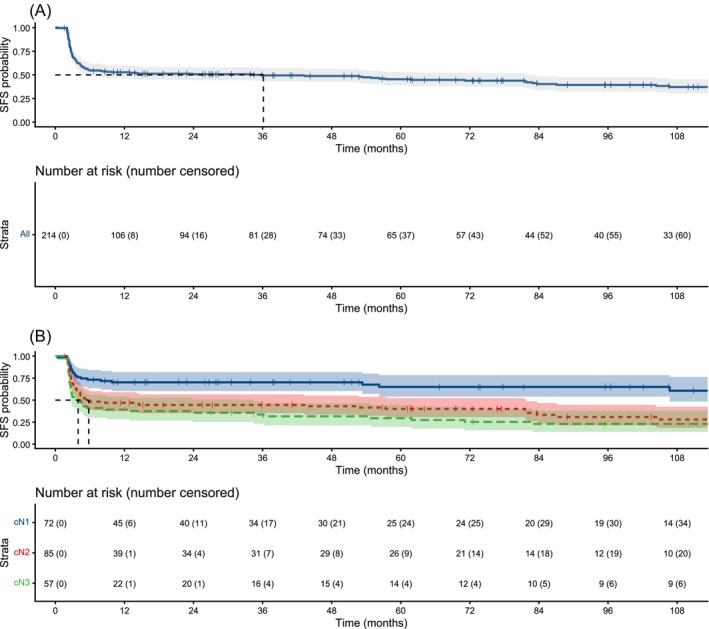
Kaplan–Meier estimated salvage chemotherapy‐free survival (SFS) curves overall after primary retroperitoneal lymph node dissection (RPLND) for clinical stage II (CS II) seminoma (A) and stratified by cN (B). Curve truncated after <10% of patients remain at risk in any single strata.

After excluding patients with presumed adjuvant treatment, 224 patients were identified who underwent pRPLND for CS II seminoma between 2004 and 2022. Median follow‐up was 11 months (IQR 2.7–73.7). Patients who received salvage treatment differed significantly in distribution of cN and pN stages from patients who remained salvage free (Table S4). Aggregate 2‐year SFS was 51%. When stratified by cN stage, patients who were cN1 had an expected SFS at 2 years of 70%. By contrast, expected 2‐year SFS for cN2 and cN3 was 45% and 36%, respectively (Figure 3). On multivariable analysis, cN2 and cN3 stage were associated with increased risk of receiving salvage treatment compared to cN1, with the hazard ratio for cN3 (HR 6.03, 95% CI 2.1–17.3, *p* = 0.00027) greater than that of cN2 (HR 3.88, 95% CI 1.87–8.03, *p* = 0.00083). pT0 stage and diagnosis between years 2017 to 2022 were also associated with increased salvage treatment, while pN0 and pN2 stage were associated with decreased salvage treatment (Table S5).

## 4 DISCUSSION

Prospective single‐arm data regarding OS after pRPLND for CS IIA/B seminoma has been excellent. The SEMS, PRIMETEST and COTRIMS trials reported OS of 100% at median follow‐up intervals of 33, 32 and 21 months, respectively.^9^, ^10^, ^11^ Contemporary retrospective series have similarly reported 100% OS at median follow‐up intervals ranging from 18.5 to 56.4 months.^6^, ^19^, ^20^ These data were concisely summarized by Kailavasan and colleagues in a 2025 review article.^21^ We demonstrate an estimated 2‐year OS of 96%. This compares favourably to those of chemotherapy and radiation, suggesting pRPLND in this setting confers at least comparable survival to these more established modalities.

A primary motivator for pRPLND is reduction of chemotherapy burden. Testicular cancer is characterized by high cure rates and young age of diagnosis, and survivors are uniquely vulnerable to the development of cardiovascular, otologic, neurologic, nephrologic and pulmonary toxicities secondary to chemotherapy or radiation exposure. Increased risk of both leukaemia and solid organ cancers has also been reported.^1^ pRPLND, when curative, eliminates the need for chemotherapy or radiation. This benefit is opposed, however, by the risk of incurring morbidity of both RPLND and chemotherapy in the event of disease relapse.

We found that pRPLND allowed approximately half of patients to avoid salvage chemotherapy. Stratification by CS showed that the benefits of pRPLND were most pronounced for patients with IIA disease, with over two‐thirds of this group able to avoid additional treatment, and that IIB and IIC disease were associated with significant stepwise decreases in SFS. This aligns with the principle that increased extent of nodal disease portends decreased likelihood of surgical cure. Indeed, it is rare to find cases of pRPLND for CS IIC disease in existing literature. The only series we identified was reported by Warszawski and Schmücking in 1997, in which 12 patients who underwent pRPLND for CS IIC seminoma experienced a 58.3% relapse rate.^4^ More interesting is that this difference in SFS exists even between CS IIA and IIB disease, as we found that patients with CS IIB seminoma were 3.88 times more likely to receive salvage chemotherapy after pRPLND than those with CS IIA. This should not be interpreted as evidence against performing RPLND for these patients, as many of these patients remained free of additional treatment after surgery. We recommend thorough counselling to impart an understanding that, while this option may be curative for CS IIB seminoma, these patients are likely at increased risk for ultimately needing additional treatment.

Our findings are directionally consistent with published recurrence‐free survival (RFS) rates from prospective trial data. Daneshmand and colleagues reported 2‐year RFS of 86% and 64% for cN1 and cN2, respectively. Hiester and colleagues reported a 2‐year RFS of 72% for a cohort consisting of about one‐third cN1 and two‐thirds cN2 disease. Finally, Heidenreich and colleagues reported a 90% RFS at 21 months median follow up for a cohort consisting of about two‐thirds cN1 and one‐third cN2 disease.^9^, ^10^, ^11^ These rates are generally better than those observed in our cohort, an effect that we propose reflects heterogeneity of care intrinsic to real‐world data that includes outcomes from lower volume centres and suggests that continued efforts to concentrate pRPLND within high‐volume centres may improve observed cure rates.

Interestingly, pN0 and pN2 pathology were significantly associated with increased SFS compared to pN1 in secondary analysis. As pN stage is understood to reflect nodal disease extent, it is unsurprising that pN0 appears protective. The signal with pN2, however, initially appears counterintuitive. Previous work has demonstrated trends towards increased recurrence with increased pN stage.^9^, ^19^ We speculate that, in this cohort, the association between pN and OS/SFS may be confounded by uncaptured surgical characteristics such as template utilization and number of nodes removed. The apparent protective effect of pN2 in this cohort might therefore reflect the effects of a more rigorous and extensive node dissection rather than the true extent of nodal disease.

### 4.1 Limitations

We acknowledge that our conclusions are limited in several respects. Our study was retrospective and is therefore subject to unaccounted confounding bias which may persist despite adjusted multivariable analysis and covariate balancing. Additionally, treatment modality is strongly associated with clinical nodal stage, making residual confounding likely and our findings ultimately hypothesis‐generating. A planned sensitivity analysis restricted to cN1–cN2 disease would further address treatment‐selection bias and will be pursued in future work.

In the absence of explicit data to distinguish planned adjuvant treatment from salvage treatment, we relied on a surrogate cutoff of 2 months from diagnosis as a discriminator. Given our intent to evaluate oncologic efficacy of pRPLND, we felt it preferable to overestimate the amount of salvage treatment received than to underestimate and report potentially inflated rates of SFS. Review of recurrence data from prospective data showed a minimum time to recurrence of 3 months.^9^, ^10^, ^11^ With this in mind, we felt that any patients who received additional treatment after pRPLND but within 2 months of diagnosis were very unlikely to have received this treatment as salvage for recurrence, hence our decision to use this as our cutoff. There nonetheless remains the possibility of some salvage treatment being considered as adjuvant and vice versa. While explicit recurrence data would have served as a valuable means to determine the nature of additional treatment, such data is not captured by the NCDB.

Other features such as surgical approach, template utilization and extranodal extension are either incompletely captured or not captured in the NCDB, therefore limiting the ability to account for their potential effects on outcomes after pRPLND. While we did not find significant differences in OS by primary treatment modality, the retrospective nature of this study precludes sample size calculations to ensure adequate powering, and there exists the possibility of small but statistically significant differences in OS that this study was not powered to detect. Finally, this data was collected prior to the availability of microRNA‐371a‐3p, a blood‐based biomarker which has shown robust ability to detect viable germ cell tumours. Ongoing efforts to combine the ability of this biomarker to detect viable disease along with pathologic confirmation via RPLND will hopefully improve identification of patients with viable retroperitoneal disease that is amenable to complete surgical resection.^22^

## 5 CONCLUSIONS

Our findings support pRPLND for CS II seminoma as a safe and potentially curative treatment in appropriately selected patients. Patients with lower volume disease benefit the most from this approach. This adds to the existing body of evidence that pRPLND should have a role in the management of CS II seminoma and provides validation against the more established approaches of primary chemotherapy and radiation.

## AUTHOR CONTRIBUTIONS

JL contributed to conceptualization, data curation, formal analysis, investigation, methodology, project administration, resources, software, supervision, validation, visualization and writing (original draft and review and editing). JM contributed to conceptualization, formal analysis, methodology, project administration, supervision, validation and writing (review and editing).

## CONFLICT OF INTEREST STATEMENT

Neither JL nor JM have any personal or financial interests to disclose. The NCDB is a joint project of the Commission on Cancer of the American College of Surgeons and the American Cancer Society. The data used in the study are derived from a de‐identified NCDB file. The American College of Surgeons and the Commission on Cancer have not verified and are not responsible for the analytic or statistical methodology employed, or the conclusions drawn from these data by the investigator. The views expressed in this article are those of the author and do not necessarily reflect the official policy or position of the Department of the Navy, Department of Defense, nor the US Government. The authors are Service members or employees of the US Government. This work was prepared as part of our official duties. Title 17, U.S.C., §105 provides that copyright protection under this title is not available for any work of the US Government. Title 17, U.S.C., §101 defines a US Government work as a work prepared by a military Service member or employee of the US Government as part of that person's official duties.

## Supporting information


**Figure S1.** Proportion of primary treatment modality for CS IIA seminoma over time.
**Table S1.** Combined table of initial univariate Cox regressions.
**Table S2.** Standardized mean differences of covariates before and after energy‐balancing weighting.
**Table S3.** Sensitivity analysis ‐ weighted multivariable Cox regression for overall survival in CSII seminoma.
**Table S4.** Baseline characteristics ‐ pRPLND seminoma only.
**Table S5.** Multivariate Cox regression ‐ secondary analysis.

## Data Availability

Per the Commission on Cancer's National Cancer Database PUF Data Sharing Agreement, the data utilized will be made available only to individuals who have applied for and been granted access to the data as a PUF Recipient. This process and the accompanying data dictionary may be accessed at https://www.facs.org/quality-programs/cancer-programs/national-cancer-database/puf/. The R code used for statistical analysis is publicly available at https://github.com/josiahlow/2025‐NCDB‐CSII‐seminoma/blob/main/ncdb_testis_csii_seminoma_pub.Rmd.
