# Supplementary material for: Primary retroperitoneal lymph node dissection for clinical stage II seminoma: Comparative analysis against established paradigms using the National Cancer Data Base
Source: BJUI Compass. 2026 May 18;7(5):e70229. doi: 10.1002/bco2.70229 (PMC13183590; doi:10.1002/bco2.70229)
Supplement: Supplementary file 1 — Figure S1. Proportion of primary treatment modality for CS IIA seminoma over time. Table S1. Combined table of initial univariate Cox regressions. Table S2. Standardized mean differences of covariates before and after energy‐balancing weighting. Table S3. Sensitivity analysis ‐ weighted multivariable Cox regression for overall survival in CSII seminoma. Table S4. Baseline characteristics ‐ pRPLND seminoma only. Table S5. Multivariate Cox regression ‐ secondary analysis. [file BCO2-7-e70229-s001.pdf]

Figure S1. Proportion of primary treatment modality for CS IIA seminoma over time

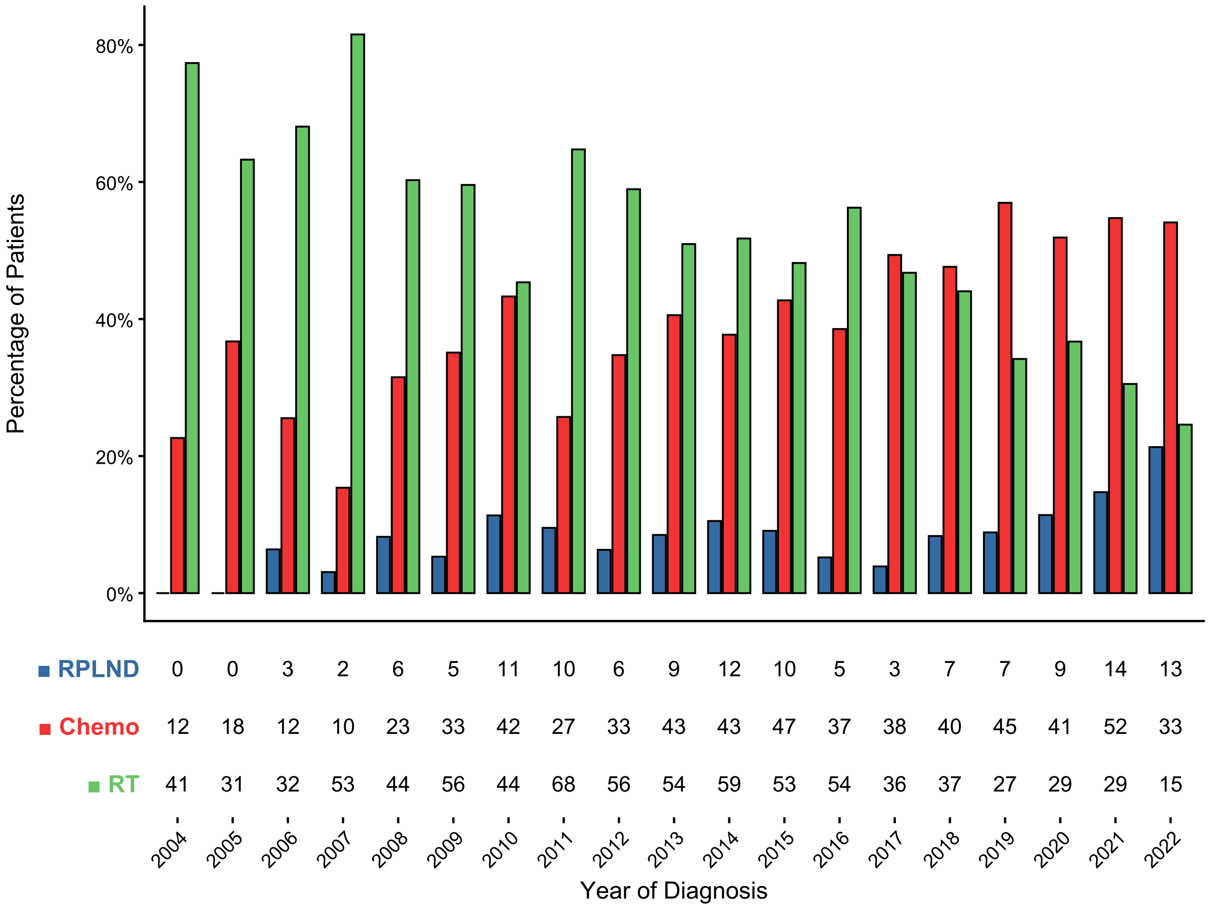

Table S1. Combined table of initial univariate Cox regressions									
		**Primary analysis:<br>OS - CSII seminoma**				**Secondary analysis: SFS - CSII seminoma,<br>primary RPLND**			
Variable		HR	LCI	UCI	p-value	HR	LCI	UCI	p-value
Age (per 1 year)		1.05	1.04	1.06	<0.0001	1.01	1.00	1.03	0.12
Year of diagnosis	2011 - 2016	1.09	0.84	1.42	0.51	1.29	0.76	2.20	0.35
(ref: 2004 - 2010)	2017 - 2022	1.25	0.90	1.73	0.18	2.29	1.35	3.91	0.0023
Charlson-Deyo score	CD1	2.20	1.58	3.06	<0.0001	1.42	0.88	2.30	0.15
(ref: CD0)	CD2	6.90	4.27	11.13	<0.0001	2.72	1.18	6.24	0.018
	CD3	3.29	1.46	7.41	0.0040	0.00	0.00	Inf	0.99
Race	Black	1.58	0.99	2.52	0.054	0.89	0.39	2.04	0.79
(ref: White)	Hispanic	0.97	0.67	1.40	0.88	1.20	0.72	2.02	0.48
	Other/Unknown	1.19	0.71	2.01	0.51	1.41	0.57	3.48	0.45
Insurance	Private	0.36	0.28	0.46	<0.0001	0.79	0.53	1.18	0.25
(ref: Medicaid/Medicare/Other Governmen	Uninsured/Unknown	0.58	0.41	0.81	0.0016	1.34	0.64	2.80	0.44
Residential category	Urban	1.20	0.89	1.63	0.23	0.76	0.41	1.41	0.38
(ref: Metropolitan)	Rural	0.99	0.41	2.41	0.99	1.11	0.35	3.49	0.86
	Unknown	1.29	0.78	2.13	0.33	0.53	0.17	1.68	0.28
Income quartile	$57,857-$74,062	1.68	1.23	2.30	0.0012	1.34	0.83	2.17	0.24
(ref: >$74,062)	$46,277-$57,856	2.15	1.58	2.94	<0.0001	1.16	0.69	1.95	0.58
	<$46,277	2.39	1.70	3.36	<0.0001	0.80	0.41	1.56	0.50
	Unknown	1.32	0.89	1.97	0.16	1.22	0.72	2.06	0.45
	5.0%-9.0%	1.32	0.94	1.86	0.11	1.38	0.80	2.37	0.24
on)	9.1%-15.2%	1.66	1.19	2.32	0.0031	1.29	0.74	2.24	0.36
(ref: <5.0%)	>15.2%	1.61	1.13	2.31	0.0091	0.82	0.43	1.57	0.56
	Unknown	1.09	0.71	1.69	0.69	1.29	0.71	2.33	0.40
d hospital	≥10 miles	0.98	0.78	1.22	0.83	0.79	0.54	1.17	0.24
(ref: <10 miles)	Unknown	0.80	0.55	1.17	0.24	0.97	0.58	1.64	0.92
Tumor size (ref: <3cm)	≥3cm	1.25	0.95	1.65	0.11	0.78	0.55	1.11	0.17
Orchiectomy margin (ref: R0)	R1	2.22	1.54	3.22	<0.0001	1.00	0.51	1.98	0.99
Pathologic T stage	pT2	1.29	1.00	1.65	0.046	1.14	0.76	1.73	0.52
(ref: pT1)	pT3	2.20	1.59	3.06	<0.0001	0.61	0.25	1.54	0.30
	pT4	5.03	2.06	12.31	0.0004	1.21	0.17	8.79	0.85
	pTis	0.00	0.00	Inf	0.99	NA	NA	NA	NA
	pT0	1.39	0.78	2.45	0.26	1.76	1.04	2.97	0.035
	pTx	1.09	0.65	1.84	0.74	1.15	0.49	2.69	0.74
Clinical N stage	cN2	1.19	0.90	1.59	0.23	2.28	1.42	3.65	.0006
(ref: cN1)	cN3	1.79	1.38	2.33	<0.0001	2.92	1.78	4.79	<0.0001
Pathologic N stage	pN2	(··)	(··)	(··)	(··)	1.24	0.71	2.15	0.44
(ref: pN1)	pN3	(··)	(··)	(··)	(··)	2.04	1.16	3.58	0.013
	pN0	(··)	(··)	(··)	(··)	0.65	0.31	1.35	0.25
Primary treatment modality	Chemotherapy	1.15	0.80	1.65	0.45	(··)	(··)	(··)	(··)
(ref: RPLND)	Radiation	0.68	0.45	1.02	0.064	(··)	(··)	(··)	(··)

Table S2. Standardized mean differences of covariates before and after energy-balancing weighting			
Covariate	Value	Standardized mean difference before energy-balancing weighting	Standardized mean difference after energy-balancing weighting
Age	(continuous)	0.33	0.03
Year of diagnosis	2004 - 2010	0.21	0.01
	2011 - 2016	0.03	0.02
	2017 - 2022	0.23	0.04
Charlson-Deyo score	CD0	0.09	0.00
	CD1	0.07	0.00
	CD2	0.01	0.00
	CD3	0.01	0.00
Race	White	0.06	0.01
	Black	0.01	0.01
	Hispanic	0.04	0.00
	Other/Unknown	0.02	0.00
Insurance	Medicaid/Medicare/Other Government	0.09	0.02
	Private	0.09	0.02
	Uninsured/Unknown	0.05	0.00
Residential category	Metro	0.03	0.00
	Urban	0.02	0.01
	Rural	0.01	0.00
	Unknown	0.02	0.00
Income quartile	>$74,062	0.07	0.01
	$57,857-$74,062	0.01	0.00
	$46,277-$57,856	0.00	0.01
	<$46,277	0.03	0.00
	Unknown	0.06	0.00
Education	<5.0%	0.05	0.00
(% without high school educa	5.0%-9.0%	0.03	0.01
	9.1%-15.2%	0.02	0.01
	>15.2%	0.06	0.00
	Unknown	0.06	0.00
Distance between	<10 miles	0.13	0.02
residence and hospital	≥10 miles	0.06	0.02
	Unknown	0.06	0.00
Tumor size	≥3cm	0.27	0.01
Orchiectomy margin	R1	0.04	0.00
Pathologic T stage	pT1	0.12	0.02
	pT2	0.06	0.00
	pT3	0.06	0.01
	pT4	0.01	0.00
	pTis	0.00	0.00
	pT0	0.08	0.03
	pTx	0.01	0.00
Clinical N stage	cN1	0.49	0.12
	cN2	0.06	0.09
	cN3	0.45	0.20

**Table S3. Sensitivity analysis - weighted multivariable Cox regression for overall survival in CSII seminoma**					
		HR	LCI	UCI	p-value
Age	(continuous)	1.05	1.04	1.07	<0.0001
Charlson-Deyo score	CD1	1.22	0.62	2.37	0.57
(ref: CD0)	CD2	6.57	3.42	12.62	<0.0001
	CD3	0.54	0.19	1.56	0.26
Insurance	Private	0.51	0.32	0.81	0.0043
(ref: Medicaid/Medicare/Other Government)	Uninsured/Unknown	0.71	0.34	1.49	0.36
Income quartile	$57,857-$74,062	1.60	0.84	3.06	0.15
(ref: >$74,062)	$46,277-$57,856	3.09	1.47	6.52	0.0030
	<$46,277	1.04	0.39	2.77	0.94
	Unknown	3.67	1.01	13.37	0.049
Education (% without	5.0%-9.0%	0.77	0.35	1.73	0.53
high school education)	9.1%-15.2%	1.81	0.80	4.09	0.16
(ref: <5.0%)	>15.2%	0.62	0.23	1.72	0.36
	Unknown	0.24	0.07	0.91	0.035
Orchiectomy margin (ref: R0)	R1	1.47	0.70	3.08	0.30
Pathologic T stage	pT2	0.92	0.57	1.50	0.75
(ref: pT1)	pT3	0.79	0.34	1.83	0.58
	pT4	1.19	0.17	8.35	0.86
	pTis	0.00	0.00	0.00	<0.0001
	pT0	0.82	0.34	1.97	0.66
	pTx	0.48	0.16	1.43	0.19
Clinical N stage	cN2	0.54	0.29	1.00	0.050
(ref: cN1)	cN3	1.59	0.89	2.84	0.11
Primary treatment modality	Chemotherapy	1.32	0.84	2.05	0.23
(ref: RPLND)	Radiation	2.31	0.97	5.50	0.058

**Table S4. Baseline characteristics - pRPLND seminoma only**				
		None/ Unknown	Salvage	p-value
		n=111	n=113	
Age (median [IQR])		44 [35-52]	43 [35-52]	0.808
Year of diagnosis	2004 - 2010	22 (20%)	17 (15%)	0.25
	2011 - 2016	46 (41%)	40 (35%)	
	2017 - 2022	43 (39%)	56 (50%)	
Charlson-Deyo score	CD0	96 (86%)	91 (81%)	0.39
	CD1	12 (11%)	18 (16%)	
	CD2	2 (2%)	4 (4%)	
	CD3	1 (1%)	0 (0%)	
Race	White	94 (85%)	86 (76%)	0.067
	Black	6 (5%)	5 (4%)	
	Hispanic	9 (8%)	17 (15%)	
	Other/Unknown	2 (2%)	5 (4%)	
Insurance	Medicaid/Medicare/Other Government	27 (24%)	28 (25%)	0.55
	Private	79 (71%)	77 (68%)	
	Uninsured/Unknown	5 (5%)	8 (7%)	
Residential category	Metro	88 (79%)	98 (87%)	0.29
	Urban	15 (14%)	10 (9%)	
	Rural	2 (2%)	3 (3%)	
	Unknown	6 (5%)	2 (2%)	
Income quartile	>$74,062	36 (32%)	37 (33%)	0.74
	$57,857-$74,062	19 (17%)	26 (23%)	
	$46,277-$57,856	19 (17%)	20 (18%)	
	<$46,277	14 (13%)	10 (9%)	
	Unknown	23 (21%)	20 (18%)	
Education	<5.0%	24 (22%)	21 (19%)	0.76
(% without high	5.0%-9.0%	23 (21%)	29 (26%)	
school education)	9.1%-15.2%	23 (21%)	28 (25%)	
	>15.2%	18 (16%)	15 (13%)	
	Unknown	23 (21%)	20 (18%)	
Distance between	<10 miles	37 (33%)	44 (39%)	0.55
residence and hospital	≥10 miles	51 (46%)	51 (45%)	
	Unknown	23 (21%)	18 (16%)	
Tumor size	<3cm	41 (37%)	57 (50%)	0.057
	≥3cm	70 (63%)	56 (50%)	
Orchiectomy margin	R0	105 (95%)	104 (92%)	0.62
	R1	6 (5%)	9 (8%)	
Pathologic T stage	pT0	5 (5%)	18 (16%)	0.057
	pTis	0 (0%)	0 (0%)	
	pT1	58 (52%)	49 (43%)	
	pT2	36 (32%)	34 (30%)	
	pT3	8 (7%)	5 (4%)	
	pT4	0 (0%)	1 (1%)	
	pTx	4 (4%)	6 (5%)	
Clinical N stage	cN1	57 (51%)	20 (18%)	<0.0001
	cN2	35 (32%)	54 (48%)	
	cN3	19 (17%)	39 (35%)	
Pathologic N stage	pN1	28 (25%)	16 (14%)	0.0020
	pN2	33 (30%)	30 (27%)	
	pN3	12 (11%)	29 (26%)	
	pN0	21 (19%)	9 (8%)	

Abbreviations: pRPLND – primary retroperitoneal lymph node dissection

**Table S5. Multivariate Cox regression - secondary analysis**					
		HR	LCI	UCI	p-value
Year of diagnosis	2011 - 2016	1.14	0.61	2.12	0.69
(ref: 2004 - 2010)	2017 - 2022	2.31	1.18	4.51	0.014
Charlson-Deyo score	CD1	1.42	0.78	2.56	0.25
(ref: CD0)	CD2	2.69	0.97	7.49	0.058
	CD3	0.00	0.00	Inf	1.0
Pathologic T stage	pT2	1.39	0.82	2.36	0.22
(ref: pT1)	pT3	0.73	0.28	1.89	0.51
	pT4	1.58	0.21	12.04	0.66
	pTis	NA	NA	NA	NA
	pT0	2.51	1.33	4.75	0.0046
	pTx	1.00	0.34	2.96	1.0
Clinical N stage	cN2	3.88	1.87	8.03	0.00027
(ref: cN1)	cN3	6.03	2.10	17.30	0.00083
Pathologic N stage	pN2	0.47	0.22	0.99	0.046
(ref: pN1)	pN3	0.43	0.15	1.29	0.13
	pN0	0.34	0.14	0.80	0.014
